# Effects of gastrointestinal motility on obesity

**DOI:** 10.1186/1743-7075-11-3

**Published:** 2014-01-07

**Authors:** Xiao-Yi Fu, Ze Li, Na Zhang, Hai-Tao Yu, Shu-Ran Wang, Jia-Ren Liu

**Affiliations:** 1 School of Public Health, JiLin Medical College, 5 JiLin street, JiLin, JiLin Province 132013, The People’s Republic of China; 2 School of Public Health, Harbin Medical University, 157 BaoJian Road, Harbin, HeiLongJiang Province 150081, The People’s Republic of China; 3 Boston Children’s Hospital and Harvard Medical School, 300 LongWood Ave, Boston 02115, USA

**Keywords:** Gastrointestinal motility, ICC, Enteric nervous system, Obesity

## Abstract

**Background:**

Changes of gastrointestinal motility, which are important related to the food digestion and absorption in the gastrointestinal tract, may be one of the factors in obesity-formation.

**Aims:**

The changes of gastrointestinal motility were explored in the rats from diet-induced obesity (DIO), diet-induced obese resistant (DR) or control (CON) by diet intervention.

**Methods:**

After fed with a high fat diet (HFD), 100 male Sprague–Dawley rats were divided into DIO, DR and CON groups. The rats from DIO and DR groups were fed with HFD, and CON with a basic diet (BD) for 6 weeks. Body weight, energy intake, gastric emptying, intestinal transit, motility of isolated small intestine segments and colon’s function were measured in this study. Expression of interstitial cells of Cajal (ICCs) and enteric nervous system (ENS) - choline acetyltransferase (ChAT), vasoactive intestinal peptides (VIP), substance P (SP) and NADPH-d histochemistry of nitric oxide synthase (NOS) were determined by immunohistochemistry.

**Results:**

Body weight and intake energy in the DIO group were higher than those in the DR group (p < 0.05). Gastric emptying of DIO group rats (78.33 ± 4.95%) was significantly faster than that of DR group (51.79 ± 10.72%) (p < 0.01). The peak value of motility in rat’s duodenum from the DR group was significantly higher than that in the DIO group (p < 0.05). In addition, the expression of interstitial cells of Cajal (ICC), choline acetyltransferase (ChAT), substance P (SP), vasoactive intestinal peptides (VIP) and neuronal nicotinamide adenine dinucleotide phosphate-diaphorase (NADPH-d) in the intestine of rats were significantly increased in the DIO group when compared to the DR group (p < 0.05).

**Conclusion:**

A faster gastric emptying, a weaker contraction of duodenum movement, and a stronger contraction and relaxation of ileum movement were found in the rats from the DIO group. It indicated that there has effect of gastrointestinal motility on obesity induced by HFD.

## Introduction

Obesity is serious public health problems in all over the world. The incidence of obesity is increasing rapidly in developed and certain developing countries. It has been reported that 30% of adults are affected by obesity in the United States [1]. It is described as a “global epidemic” by the World Health Organization (WHO) paralleled by a dramatic rise in the prevalence of diabetes over the past 30 years [2]. Obesity is a serious disease because it is a risk factor for cancer, type 2 diabetes, hypertension, cardiovascular disease and other chronic diseases. Pharmacotherapy, behavior and dietary modification could temporal alleviate the symptoms of obesity [3,4]. Surgical treatments are the most effective way for obesity, which result in substantial weight loss. However, the limitations of these surgeries were noted due to their complications and morbidities [5]. Thus, there are no effective ways to therapy obesity [6].

The gastrointestinal (GI) tract is an important organ in regulating food-taking, digestion and absorption of nutrients. Available data show that absorption of nutrients in small intestine can be affected by the alteration of intestinal motility in humans and rats [7-11]. A previous study showed that the changes of gastrointestinal motility occurred in the dog’s study [12]. An extension of luminal transit time has relationship with a linear increase of the absorption of nutrients. Thus, whether there are the changes of GI motility in obesity may provide a useful target for obesity-treatment.

Interstitial cells of Cajal (ICCs) - mediators of neuromuscular transmission in the gastrointestinal tract, which express c-kit receptor tyrosine kinase, are identified by Cajal SR in 1893 [13]. ICCs are pacemaker cells for the gastrointestinal movement, and play crucial roles in the regulation of GI motility [14-16]. Two classes of ICCs have been recognized in the stomach of mammalian: myenteric ICCs (ICC-MY) and intramuscular ICCs (ICC-IM). ICC-MY, which lie between the circular and longitudinal muscles in the region around the myenteric plexus, have been identified as the source of the electrical slow waves underlying the phasic contractions of the gastric musculature [17,18]. ICC-IM, which are observed in the circular and longitudinal muscle layers, can play a role in mediating excitatory and inhibiting inputs to the musculature from the enteric motor neurons [19,20]. A previous study demonstrated that almost ICCs express c-kit, all c-kit cells expressed by gastrointestinal tract are ICCs. Thus, c-kit can be a marker to identify ICCs for the study of GI motility [21]. In addition, a critical controller of gastrointestinal functions - enteric nervous system (ENS), is independent of the central nervous system [22]. ENS is an integrated neuronal network localized along the gastrointestinal tract and organized in two major plexuses: the myenteric and the submucosal plexuses [23]. Myenteric neurons are mainly involved in the control of GI motility. The motor neurons innervating circular muscle consist of two main classes of myenteric neurons which have distinct functions, descending inhibitory neurons-containing neuromediators or enzymes such as vasoactive intestinal peptides (VIP) and nitric oxide synthase (NOS); and ascending excitatory neurons-containing choline acetyltransferase (ChAT) and substance P (SP) [24,25]. Therefore, the complicated mode of ENS functions in the GI tract provides us a potential target for obesity.

The objectives of this study were to determine that: (1) diet–induced obesity (DIO) and diet-induced obesity resistance (DR) rats were induced by high fat diet (HFD); (2) the changes of gastrointestinal motility were measured in DIO and DR groups; (3) the changes of serum gastrointestinal hormones such as cholecystokinin (CCK), motillin and gastrin were examined in DIO and DR groups; and (4) the possible mechanism of a regulating gastrointestinal motility was related to the cells and neurons in the GI tract.

## Materials and methods

### Experimental design and animal care

A total of 100 male Sprague–Dawley rats, body weight 180 ± 10 g, purchased from the Beijing Vital River Laboratories Animal Research Center (Animal license No. SCXK (Jing) 2006–0009). The rats were housed in a temperature-controlled room (22 ± 1°C) and a relative humidity of 60% with a 12-h light/12-h dark cycle and water was available ad libitum. The rats were housed individually in the wire cages and acclimated to the surroundings in the animal room for one week. All animal experiments were carried out in accordance to the guidelines of China legislations on the ethical use and care of laboratory animals. All experimental procedures were approved by the Animal Ethics Committee, JiLin Medical College (JiLin, China).

**Figure 1:**
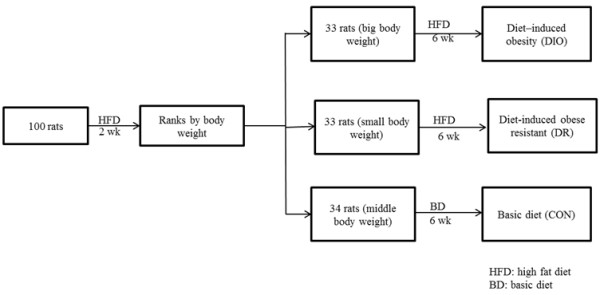
The study design and the flow of treatment groups and times.

The rats were fed with a purified American Institute of Nutrition (AIN-93G) diet as a basic diet (BD). A high-fat diet (HFD, 5.26 kcal/g) based on a basic diet modified containing 59.4% fat, 19.8% carbohydrates and 20.8% protein (Tables 1 and 2). A total of 100 rats were fed with HFD for two weeks. The rats were sorted according to their body weight. Thirty-three rats with the highest gain of body weight, which were considered susceptible to obesity, assigned to as a diet–induced obesity (DIO). Thirty-three rats with the lowest gain of body weight, which were considered resistant to obesity, assigned to as a diet-induced obese resistant (DR) and the rest rats as a control group (CON) (Figure 1). Rats from DIO and DR groups were fed with HFD for 6 weeks and the rats in the CON group with BD. Body weight was measured weekly. Food take was measured every 24 h for calculation of energy intake (kcal) by multiplying the food weight (g) and energy density. At experimental termination, ten rats from each group were sacrificed under anaesthetized with sodium pentobarbital (40 mg/kg, ip). The blood from rats was collected for determination of serum gastrointestinal hormones. Segments of duodenum (approximately 2 cm on the anal side from the pylorus ring), jejunum (approximately 2 cm on the anal side from the duodenojejunal flexure) and ileum (approximately 2 cm on the oral side from the cecum) regions were collected for the measurement of motility of intestine segments or immunohistochemical staining. Ten rats from each group were to determine gastric emptying. The rest ten rats from each group were determined the colon’s function as below.

**Table 1:** Composition of purified experimental diets (g/kg diet)

**Components**	**High fat diet (g)**	**Basic diet (g)**
Corn starch	130	330
Casein	270	220
Dextrin	65	165
Soybean oil	64	48
Lard	283	4
Sucrose	65	165
Cellulose	27	20
Mixing minerals	48	35
Mixing vitamines	14	10
L-cystine	4	3
Bile salts	2.7	2

Mixing mineral: CaHPO_4_, NaCl, K_3_C_6_H_5_O_7_·H_2_O, K_2_SO_4_, MgO, MnCO_3_, FeC_6_H_5_O_7_·5H_2_O, ZnCO_3_, CuCO_3_·Cu(OH)_2_·H_2_O, KIO_3_, Na_2_SeO_3_·5H_2_O and CrK(SO_4_)_2_·12 H_2_O; Mixing vitamins: vitamin A, vitimins B1, B2, B6, B12, vitamin D3, vitamin E, vitamin K3, nicotinic acid, pantothenic acid, folic acid and biotin.

**Table 2:** Energy provided by protein, fat, and carbohydrate of experimental diets

**Diet**	**Protein (%)**	**Fat (%)**	**Carbohydrates (%)**	**Total energy (kJ/g diet)**
Basic	20.7	11.9	67.3	16.4
High fat	20.8	59.4	19.8	22.0

### Gastric emptying

Gastric emptying was determined according to a previous method [26]. Briefly, the stomachs from 10 rats in each group were gavaged 3 mL of 0.05% phenol red solution (Sigma Chemical Co., St. Louis, MO) and administered 20 min before executing the rat. The stomach from each rat was removed after ligation of both the cardiac and pyloric ends, and placed in 100 mL of 0.1 mol/L of NaOH. The entire stomach including its content was homogenized for 30 s at medium speed used by tissue disperser machine (IKA, Germany), and then the mixture was kept for 1 h at room temperature. Five mL supernatant was added to 1 mL 33% trichloroacetic acid and centrifuged at 2500 g for 20 min. After centrifuged, the supernatant was added 4 mL 0.5 mol/L NaOH to develop the maximum intensity of the color. The absorbance of each sample was measured at 560 nm with a spectrophotometer. Liquid gastric emptying (GE) for each rat was calculated with the following formula:

$$\mathrm{Gastric}\;\mathrm{emptying}\left(\%\right)=\left(1-\mathrm{absorbance}\;\mathrm{of}\;\mathrm{test}\;\mathrm{sample}/\mathrm{absorbance}\;\mathrm{of}\;\mathrm{baseline}\;\mathrm{control}\right)\times 100\%.$$

### Intestinal transit

After the excision of the stomach, the entire small intestine was removed from its mesenteric attachments immediately, and its length was measured from the pyloric sphincter to the ileocecal junction. The phenol red could be shown by a few drops of 0.1 mol/L NaOH. This method could be used to determine the traveled distance of phenol red. Intestinal transit for each rat was calculated with the following formula:

$$\mathrm{Intestinal}\;\mathrm{transit}\;\left(\%\right)=\left(\mathrm{the}\;\mathrm{distance}\;\mathrm{traveled}\;\mathrm{by}\;\mathrm{the}\;\mathrm{test}\;\mathrm{liquid}/\mathrm{the}\;\mathrm{total}\;\mathrm{length}\;\mathrm{of}\;\mathrm{intestine}\right)\times 100.$$

### Examination of colon’s function - fecal output

The fecal output was examined according to a previous study [27]. Ten Rats from each group were placed in individual metabolism cages with free access to food and water. The rats were acclimatized to the same controlled condition for 3 d before this experiment. During this period, the rats were only fed with HFD (5.26 kcal/g) and ad libitum access to water. The fecal output excreted by each rat during a period of 8, 12 and 24 h were collected and marked wet fecal weight immediately and dry fecal weight after drying (24 h at 46°C). The secretion of colon was expressed as the ratio of wet fecal weight to dry fecal weight.

### Motility of isolated small intestine segments

The motility of isolated small intestine segments was examined according a previous study [28]. Briefly, segments of duodenum and ileum regions immediately placed vertically in 10 mL baths individually installed in tensity transducer (RM6240 Multi-channel physiological signal acquisition and processing system) filled with Krebs-Henseleit solution (NaCl, 118 mmol/L; KCl, 4.7 mmol/L; CaCl_2_, 2.5 mmol/L; KH_2_PO_4_, 1.2 mmol/L; NaHCO_3_, 25 mmol/L; MgSO_4_, 1.2 mmol/L; and glucose, 10.0 mmol/L) at the temperature of 37°C with air circulation (O_2_, 95%; CO_2_, 5%). The distal end of the excised intestinal section was fixed to the bottom of the bath and the other end was fixed to the tension-measuring instrument. The tissues were then allowed to equilibrate for at least 30 min, during which time the tension was adjusted to maintain a 1 g stable resting tension during the experiment period. The frequency of recorded isometric tension of intestinal section was measured for approximately 30 min. All recorded values were calculated with the software of RM6240.

### Serum gastrointestinal hormones

Serum gastrointestinal hormones including cholecystokinin (CCK), motillin and gastrin were measured by using ELISA kits (Phoenix Pharmaceuticals, Burlingame, CAUSA) and following the manufactory’s instruction [29].

### Immunohistochemistry staining

Isolated rat duodenum, jejunum and ileum from BIO and DR groups were fixed with 4% paraformaldehyde and then, dehydrated in 5 and 15% glucose gradient for 1 h, and stayed overnight in 30% glucose gradient in 4°C. All tissues were embedded with optimum cutting temperature medium after overnight and kept for test in -80°C. All issues were cut along the short axis into 7 μm. The slices were incubated with primary antibodies overnight at 4°C. These primary antibodies were as follows: rabbit anti-c-kit polyclonal antibody (1:100, sc-168), or rabbit anti-choactase (H-95) polyclonal antibody (1:50, sc-20672); or anti-VIP (H-95) polyclonal antibody (1:50, sc-20727), or goat anti-Substance P (E-15) polyclonal antibody (1:50, sc-14104, Santa Cruz Biotechnologies, CA, USA). After washing with PBS, these slices incubated with anti-rabbit or anti-goat second antibodies at 37°C for 30 min. After the chromogenic reaction was developed with 3, 3′-diaminobenzidine (DAB) for 1 min, all sections were counterstained with hematoxylin. The same protocol was applied to the negative control with the omission of the primary antibody. Images were captured under microscope with a camera (Olympus, Japan) and analyzed with a microscope (Nikon 55I, Tokyo, Japan). The integral optical density (IOD) of positive cells was quantified by image analysis software IPP6.0 as a previous study [30].

### NADPH-d histochemistry of NOS

For beta-NADPH immunohistochemistry, slides of each intestinal section mentioned above were incubated in 1 mg/mL of beta-NADPH (Sigma Chemical Co.), 0.25 mg/mL nitro blue tetrazolium (Sigma Chemical Co.), and 0.3% Triton X-100 in 0.05 mol/L Tris–HCl buffer (pH 7.6) at 37°C for 2 h, and then mounted using glycerol mounting medium according a previous study [31].

### Statistical analysis

Data was expressed as means ± SEM (standard error of mean). Statistical analysis was performed using SPSS 20.0 (SPSS Inc., Chicago, IL). The data of normality and equal variance were tested using the Kohnogorov-Smirnov test and the Levene Median test, respectively. The data comparison in groups was analyzed using Student’s *t* test, Welch’s *t* test, or ANOVA. Statistical significance was set at p < 0.05 and p < 0.01, and all *P* values were unadjusted for multiple comparisons.

## Results

### Body weight and energy intake

**Figure 2:**
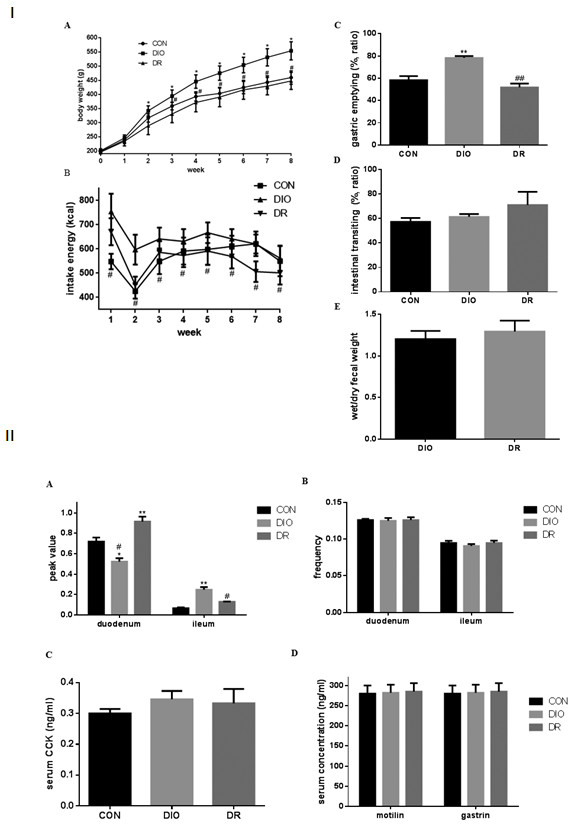
**The changes of body weight and gastrointestinal motility as well as the changes of isolated small intestine segments and serum gastrointestinal hormones in food-interfered and control groups. I. A** body weight; **B** intake of energy; **C** the ration of gastric empty in CON, DIO and DR groups. **D** the rate of intestinal transiting in CON, DIO and DR groups. **E** the ratio of wet/dry fecal weight in DIO and DR groups. **II. A** the summary of peak changes of duodenum and ileum; **B** the summary of frequency changes of duodenum and ileum; **C** the changes of serum cholecystokinin (CCK); **D** the changes of serum motillin and plasma gastrin. CON: the group of basic diet control; DIO: the group of diet–induced obesity; DR: the group of diet-induced resistance. *p < 0.05, **p < 0.01, compared to the CON group. #p < 0.05, ##p < 0.01, compared to the DIO group.

A total of 100 male SD rats were fed with high fat diet (HFD) for two weeks. The rats were divided into 3 groups according to their body weight (Figure 1). The rats were fed with HFD or basic diet (BD) for six weeks (Figure 1). The body weight in each group did not have difference in the initial and first week of animals (Figure 2I A). After 1^st^ week, the body weight of rats in the DIO group was significantly increased when compared to the CON group (p < 0.05). After 2^nd^ week, the body weight of rats in the DIO group also showed a significant increase in comparison with the DR group (p < 0.05). No differences were found between DR and CON groups during six weeks feeding.

In this study, food intake also was measured and energy intake (kcal) was calculated in these rats. The results showed that intake energy in the DIO group was significantly higher than that in the DR group (p < 0.05) (Figure 2I B). However, there was no differences between DIO and CON groups (p > 0.05).

### Gastric emptying, gastrointestinal transit and fecal output

As shown in Figure 2I C, the gastric emptying was significantly highly in the DIO group (78.33 ± 4.95%) when compared to the CON (58.32 ± 11.61%) and DR (51.79 ± 10.72%) groups (p < 0.05). No differences were found between CON and DR groups (p > 0.05). To determine gastrointestinal transit, the distance traveled by the phenol red from the pyloric sphincter to the ileocecal junction was measured in this study. The results are summarized in Figure 2I D. The gastrointestinal transit did not have differences in each group (p > 0.05). In addition, to examine colon’s function, fecal output was measured in DIO and DR groups. The fecal wet and dry mass were 1.20 ± 0.31 and 1.29 ± 0.42 in DIO and DR groups, respectively. No differences were found between DIO and DR groups (Figure 2I E).

### Motility of the duodenum and ileum, and serum gastrointestinal hormones

In order to determine the motility of small intestine, segments of duodenum and ileum regions were isolated and tension of intestinal muscle was measured in this study. The results are shown in Figure 2II. The peak values of duodenum were significant higher than that in the ileum (Figure 2II A). The peak values of duodenum were significantly increased in DR group when compared to the CON group (p < 0.01). A significant decreasing peak values of duodenum was shown in the DIO group in comparison with the CON group (p < 0.05). The peak values of ileum were the highest in the DIO group and the lowest in the CON group (Figure 2II A). They had significant differences amongst CON, DIO and DR groups (p < 0.05 or p < 0.01). In addition, the frequencies of constriction in duodenum and ileum were also measured in this study. The results are shown in Figure 2II B. Although the frequency of constriction in duodenum was higher than that in ileum, no differences were found amongst CON, DIO and DR groups.

Gastrointestinal hormones play important roles in appetite and eating [32]. Serum gastrointestinal hormones such as CCK, motilin and gastrin were examined in this study. As shown in Figure 2II C-D, the concentrations of CCK, motilin and gastrin did not difference in rat sera amongst CON, DIO and DR groups. It seems that secretion of gastrointestinal hormones do not affect the rat’s appetite and eating in this study.

### Expression of interstitial cells of Cajal (ICC) small intestine

**Figure 3:**
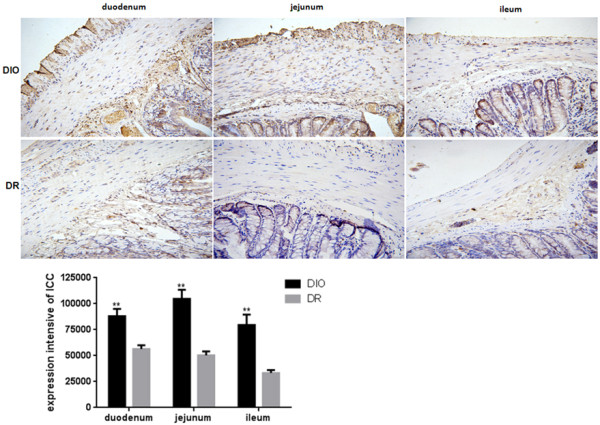
**Expression of interstitial cells of Cajal (ICC) in the duodenum, jejunum and ileum of rats from DIO and DR groups (n = 6).** ICC was determined by an antibody against c-kit. Positive cells of C-kit-in the circular muscle and myenteric plexus are shown the brown color and designated as ICC-IM and ICC-MY, respectively. Integral optical density (IOD) of positive ICC expression was quantified by image analysis. DIO: the group of diet–induced obesity; DR: the group of diet-induced resistance. Scale bar = 50 μm. **p < 0.01, compared to the DIO group.

ICC is a pacemaker which connected intestinal cells with the rhythmicity of gut motor activity [33]. ICC amount is an important factor for gut motor activity. In our study, expression of ICC was examined in duodenum, jejunum and ileum of rats fed with HFD. The results are shown in Figure 3. The expression of ICC in duodenum, jejunum and ileum of rats was significant higher in DIO group than those in DR group (p < 0.01). The maximum integral optical density (IOD) of positive ICC was found in jejunum of rats and following by duodenum and ileum. The expression of ICC in DIO group was increased 1.6-, 2.1- and 2.4-fold in duodenum, jejunum and ileum, respectively, when compared to the DR group.

### Expression of ChAT, SP, VIP and NADPH-d histochemistry of NOS in intestine

**Figure 4:**
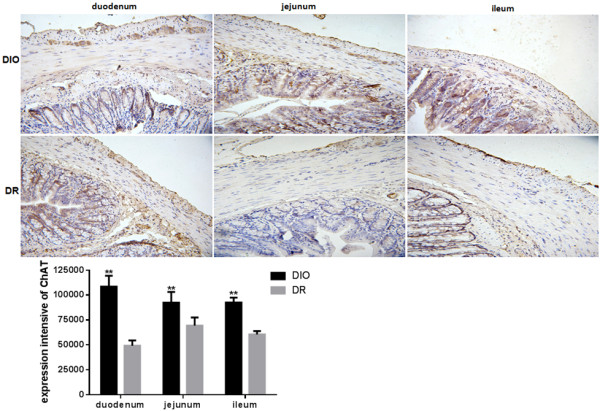
**Expression of enteric nervous system (ENS)-choline acetyltransferase (AchE) in the duodenum, jejunum and ileum of rats from DIO and DR groups (n = 6).** Myenteric neurons were stained with anti-ChAT antibody in the duodenum, jejunum and ileum of rats. A brown color is shown the expression of ChAT in neurons. Integral optical density (IOD) of positive ChAT expression immunoreactive neurons were quantified by image analysis. DIO: the group of diet–induced obesity; DR: the group of diet-induced resistance. Scale bar = 50 μm. **p < 0.01, compared to the DIO group.

**Figure 5:**
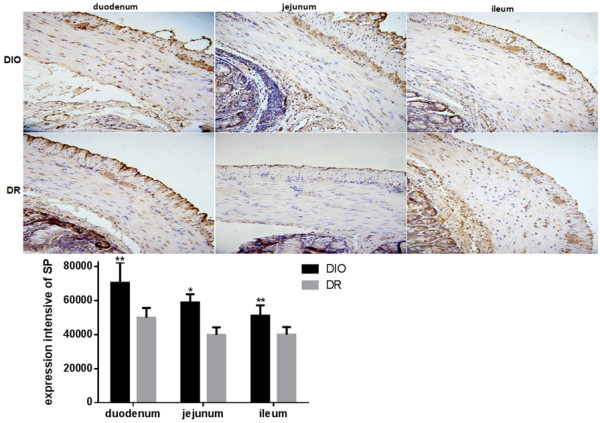
**Expression of substance P (SP) in the duodenum, jejunum and ileum of rats from DIO and DR groups (n = 6).** Expression of SP in duodenum, jejunum and ileum of rats was examined by immunohistochemistry. Positive expression of SP showed a brown color. Integral optical density (IOD) of positive SP expression was quantified by image analysis. DIO: the group of diet–induced obesity; DR: the group of diet-induced resistance. Scale bar = 50 μm. **p < 0.01, compared to the DIO group.

To further determine the relationship between intestinal nervous system and diet intervention, expression of excitatory neurons and inhibitory neurons was determined in the intestinal wall of rats. The distribution and regional variation of acetylcholinesterase (AChE), and substance P (SP), vasoactive intestinal peptides (VIP) and nitric oxide synthase (NOS) were investigated in the duodenum, jejunum and ileum of rats. As a selective marker of cholinergic neurons, the expression of choline acetyltransferase (ChAT) was examined in duodenum, jejunum and ileum of rats. As shown in Figure 4, expression of ChAT in duodenum, jejunum and ileum of rats was significant higher in the DIO group than those in the DR group (p < 0.01). The expression of ChAT in the DIO group was increased 2.2-, 1.3- and 1.5-fold in duodenum, jejunum and ileum, respectively, when compared to the DR group. Another marker for sensory innervations, the expression of SP also was determined in this study. The results showed that the expression of SP in duodenum, jejunum and ileum of rats was significant higher in the DIO group than those in the DR group (p < 0.01) (Figure 5). The SP expression in the DIO group had 1.4-, 1.5- and 1.3-fold increase in duodenum, jejunum and ileum, respectively, in comparison with the DR group.

**Figure 6:**
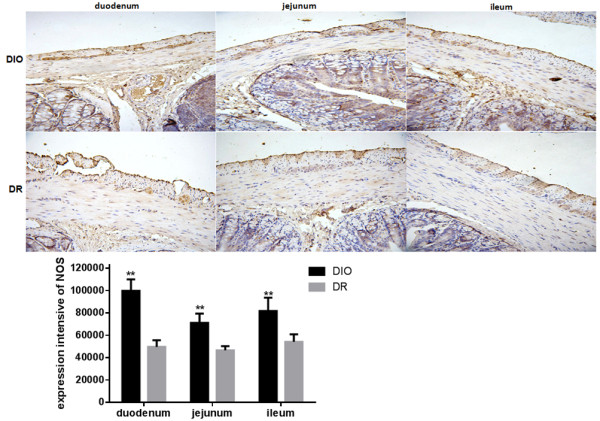
**Expression of NADPH-d histochemistry of nitric oxide synthase (NOS) in the duodenum, jejunum and ileum of rats from DIO and DR groups (n = 6).** Integral optical density (IOD) of positive NOS expression was quantified by image analysis. DIO: the group of diet–induced obesity; DR: the group of diet-induced resistance. Scale bar = 50 μm. **p < 0.01, compared to the DIO group.

**Figure 7:**
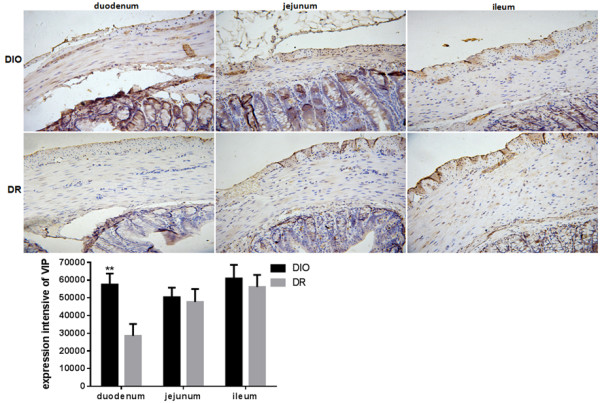
**Expression of vasoactive intestinal peptides (VIP) in the duodenum, jejunum and ileum of rats from DIO and DR groups (n = 6).** Expression of VIP in the duodenum, jejunum and ileum of rats was examined by immunohistochemistry. Positive expression of VIP showed a brown color. Integral optical density (IOD) of positive VIP expression was quantified by image analysis. DIO: the group of diet–induced obesity; DR: the group of diet-induced resistance. Scale bar = 50 μm. **p < 0.01, compared to the DIO group.

In addition, nitric oxide as an inhibitory nonadrenergic non-cholinergic neurotransmitter is expressed in the gastrointestinal tissue [34]. NADPH-d (Neuronal nicotinamide adenine dinucleotide phosphate-diaphorase) which is a nitric oxide synthase (NOS) may be as a marker for neurons producing nitric oxide. In this study, the NOS expression was examined in the duodenum, jejunum and ileum of rats. As shown in Figure 6, expression of NOS in the duodenum, jejunum and ileum of rats was significant higher in the DIO group than those in the DR group (p < 0.01). NOS expression in the DIO group had 2.0, 1.5- and 1.5-fold increase in the duodenum, jejunum and ileum, respectively, in comparison with the DR group. In this study, expression of VIP also was determined in the duodenum, jejunum and ileum of rats. The results are shown in Figure 7. The expression of VIP in duodenum of rats was significant higher in the DIO group than those in the DR group (p < 0.01). The VIP expression in the duodenum was increased to 2.0-fold in the DIO group when compared to the DR group (p < 0.01). No differences were found the expression of VIP in the jejunum and ileum of rats between DIO and DR groups.

## Discussion

Gastrointestinal (GI) tract plays the functions of digestion and absorption, which is important to the regulation of energy homeostasis. Gastric emptying is defined as the process of transforming food from the stomach into the duodenum. A fast speed of gastric emptying can result in a short time of fullness and being easy to feel hungry. In this study, rats with diet-induced obesity (DIO) or diet-induced obese resistant (DR) were screened from 100 SD male rats fed with high fat diet (HFD) for two weeks. The rats in the DIO group had a fast gastric emptying when compared to the DR or CON groups. The food-intake and intake energy of rats in the DIO group was much more than those of DR group. Body weight of rats in the DIO group were increased when compared to the DR or CON groups. Previous studies have been shown that the shape, properties, composition, energy density and total energy were correlated with the speed of gastric emptying [35,36]. It suggests that gastric emptying is one of predominated factors of obese formation in the rats fed high fat diet. In addition, the measurement of intestinal transit and colon’s function was followed by gastric empting. The result of intestinal propulsion showed that the distance of liquid propulsion in the DIO group was smaller than that in the DR group. However, there did not have differences between DIO and DR groups. Fecal excretion can be considered as an index of the function of colonic transiting. Our results showed that the ratio of wet to dry fecal weight did not have differences between DIO and DR group. Thus, gastrointestinal transit and fecal output do not related to the obese rats fed with HFD in this study.

The GI tract, unlike other organs, is able to fulfill the functions of motility even when isolated from the body. It is necessary to measure the motility of the isolated small intestine-duodenum and ileum intestinal section. In this study, the peak values of duodenum were significantly decreased and the peak values of ileum were significantly increased in the DIO group when compared to the CON group. However, opposite results of peak values in the duodenum and ileum were found in the DR group in comparison with the DIO group. It means that the contract and relaxation of rat duodenum in the DIO group were weaker than that in the DR group. In the rat ileum, the stronger contraction and relaxation, the more stability of tension, the same speed of ileum movements and the more stability of speed were found in the DIO group. As known, compared to the ileum, duodenum mixes the gastric juice, pancreatic juice and bile, and plays a particularly prominent role in the function of digestion. The food clumps emptied from the stomach can be digested completely into small molecules which can be easily absorbed in duodenum [37,38]. A more efficient absorption of nutrients definitely exists in the proximal intestine - duodenum other than ileum [39]. It has been demonstrated that only small amounts of unabsorbed nutrients may transit into the ileum. In this study, the weaker contract and relaxation and the smaller speed of movement existing in the duodenum in the DIO group may result in a long term staying of food in the duodenum in order to digest the food more completely and easier to be absorbed. Thus, weight gain of rats was much more in the DIO group in comparison with the DR group.

In our previous study, improving chewing activity could reduce energy intake and modulate plasma gut hormone concentrations in obese and lean young Chinese men [29]. However, in our study, the levels of gastrointestinal hormones such as CCK, motillin and gastrin in rats were not different between DIO and DR groups. It indicated that regulation of gastrointestinal hormones does not predominate in the rats from the DIO and DR groups fed with HFD. To further explain the mechanism of intestinal motility, interstitial cells of Cajal (ICC) in small intestine was determined. ICC, a kind of special mesenchymal cell in gastrointestinal tract and as the pacemaker cells of gastrointestinal motility and intermediary controller of neural of intestinal muscle, plays a series roles in not only slowing wave activity of gastrointestinal involved in slow wave conduction, but it also regulates neuromuscular signal conduction [33,40,41]. In a previous study, almost all ICCs could express the c-kit and almost all ICCs in gastrointestinal tract express ICCs [33]. Thus, c-kit can be a marker to identify ICCs. In this study, the amount of c-kit expression in the duodenum, jejunum and ileum of rats was significantly high in the DIO group when compared to the DR group. It suggests that there have more ICCs in rat gastrointestinal tract from the DIO group to enhance the motility of GI.

In addition, in order to explain the intestinal motility, enteric nervous system (ENS) also was examined in this study. ENS is the specific nervous system which is able to make intestine to fulfill its motility function of GI which is also closely related to ICCs, ENS cells have synapse-like connections with ICC-IM cell bodies and gap-like junctions with smooth muscle cells [42]. These specific structural basis make it possible that ICCs may be dominated by ENS through the way of transmission and regulation of various neurotransmitters and regulate GI smooth muscle contraction through the bridge role of ICCs existing in ENS and smooth muscle [43]. The GI motility patterns were formed by the integration between the ENS and ICCs system (including the pacing ability of ICC-MY and the media role of ICC-IM). In ENS, especially in myenteric nerve plexus, there are stimulating neurons - choline acetyltransferase (ChAT) and substance P (SP) which respectively produce excitatory neurotransmitter – acetylcholine (Ach) and SP to make intestinal muscle contraction, and inhibitory neurons - nitric oxide synthase (NOS) and vasoactive intestinal peptides (VIP) which respectively produced inhibitory neurotransmitter - nitric oxide (NO) and VIP to make intestinal muscle relaxation [44,45]. In this study, both stimulating and inhibitory neurons - ChAT, SP, NOS and VIP were significantly high expression in the DIO group in comparison with the DR group. These suggest that the contraction and relaxation - intestinal motility regulated by ENS in the rats from the DIO group were stronger than those in the DR group. In a previous study, the nutrients absorbed by GI tract had interacted with neurotransmitter produced by ENS, they enforced the functions of each other [46]. The weaker motility in the duodenum and stability in all intestinal sections provided the process of food-digestion by duodenum and food-absorb by intestinal villus. Thus, neurotransmitters produced by ENS could promote the GI motility and enhance absorption of nutrients in high fat diet in this study.

In summary, rats were screened to diet-induced obesity (DIO) or diet-induced obese resistant rats (DR) from the rats fed with HFD for two weeks. A faster gastric emptying, a weaker contraction of duodenum movement, and a stronger contraction and relaxation of ileum movement were found in the rats from the DIO group when compared to the DR group. These changes of gastrointestinal motility might be regulated by increasing ICC amount, enhancing excitatory neurons and inhibitory neurons of enteric nervous system in the DIO group. However, no differences were found in the levels of serum gastrointestinal hormones such as CCK, motillin and gastrin. It suggested that diet intervention plays an important role in gastrointestinal motility, especially, in formation of obesity.

## Competing interests

The authors declare that they have no competing interests.

## Authors’ contribution

SRW, ZL and XYF designed the research. XYF, ZL and NZ conducted the animal experiment. XYF, ZL and HTY conducted the experimental procedure such as immunohistochemistry, and functional measurements. SRW, JRL and XYF analyzed the experimental data and interpretation the data. SRW and JRL wrote the drafting of the manuscript and final approved the manuscript. All authors read and approved the final manuscript.
